# Variant-specific Symptoms After COVID-19: A Hospital-based Study in Hiroshima

**DOI:** 10.2188/jea.JE20230103

**Published:** 2024-05-05

**Authors:** Kanon Abe, Aya Sugiyama, Noriaki Ito, Kei Miwata, Yoshihiro Kitahara, Mafumi Okimoto, Ulugbek Mirzaev, Akemi Kurisu, Tomoyuki Akita, Ko Ko, Kazuaki Takahashi, Tatsuhiko Kubo, Toshiro Takafuta, Junko Tanaka

**Affiliations:** 1 Department of Epidemiology, Infectious Disease Control and Prevention, Graduate School of Biomedical and Health Sciences, Hiroshima University, Hiroshima, Japan; 2 Hiroshima City Funairi Citizens Hospital, Hiroshima, Japan; 3 Department of Hepatology, Scientific Research Institute of Virology, Ministry of Health of Uzbekistan, Tashkent, Uzbekistan; 4 Department of Public Health and Health Policy, Graduate School of Biomedical and Health Sciences, Hiroshima University, Hiroshima, Japan

**Keywords:** post COVID-19 condition, long COVID, variant, risk factor, Japan

## Abstract

**Background:**

Symptoms after novel coronavirus disease 2019 (COVID-19) recovery by severe acute respiratory syndrome coronavirus 2 strains are unspecified.

**Methods:**

This self-administered questionnaire-based study was conducted to investigate symptoms after COVID-19 recovery at one of the main hospitals for COVID-19 treatment in Hiroshima, Japan, from September 2020 to March 2022 for patients who visited follow-up consultations after COVID-19. Study subjects were divided into four groups (Wild-type, Alpha, Delta, and Omicron periods) according to COVID-19 onset date. Hierarchical cluster analysis was performed to determine symptom clusters and investigate risk factors for each symptom cluster using multivariate analysis.

**Results:**

Among 385 patients who enrolled in this study, 249 patients had any persistent symptoms at a median of 23.5 (interquartile range, 20–31) days after COVID-19 onset. Among patients with any persistent symptoms, symptom clusters including olfactory or taste disorders, respiratory symptoms, and cardiac symptoms were found. Respiratory symptoms were more frequent among patients infected in the Omicron period compared to the Wild-type period (adjusted odds ratio [AOR] 3.13; 95% confidence interval [CI], 1.31–7.48). Compared to patients who recovered from mild COVID-19, patients who needed oxygen or ventilation support suffered fewer post-COVID-19 respiratory symptoms (AOR 0.46; 95% CI, 0.22–0.97) but more post-COVID-19 cardiac symptoms among them (AOR 2.67; 95% CI, 1.26–5.65). Olfactory or taste disorders were fewer among patients infected in the Omicron period compared to the Wild-type period (AOR 0.14; 95% CI, 0.04–0.46).

**Conclusion:**

This study revealed that symptoms after COVID-19 may vary depending on the infected strain.

## INTRODUCTION

The severe acute respiratory syndrome coronavirus 2 (SARS-CoV-2) is responsible for the novel coronavirus disease 2019 (COVID-19). The virus undergoes gradual mutations from its original form, also known as the Wild-type, resulting in variants of concern (VOC) that exhibit increased transmissibility, virulence, and pathogenicity, as well as decreased effectiveness of diagnostic, prevention, and therapeutic measures.^1^ The World Health Organization (WHO) has established molecular surveillance of mutations since the beginning of the pandemic, identifying five VOCs, namely Alpha, Beta, Gamma, Delta, and Omicron.^2^

COVID-19 patients have a variety of symptoms, with differing disease severity among individuals. Additionally, patients may encounter diverse long-term effects after recovery.^3^^–^^5^ These effects have been given various labels, including long COVID,^4^ post COVID-19 condition,^4^ post-COVID conditions,^5^ and post-COVID-19 syndrome.^3^ There are reports suggesting that these post-COVID-19 symptoms may persist for a year or more,^6^^,^^7^ adding to the complexity of this disease.

In the guideline for the clinical management of patients with COVID-19 in Japan, post-COVID-19 symptoms have not been well defined yet, and its pathogenesis is still unclear.^8^ These symptoms can cause physical, mental, and neurological effects, reducing the quality of life of patients after recovery from COVID-19.^9^^,^^10^ Although previous studies have indicated that the duration between SARS-CoV-2 infection and disease onset and clinical symptoms varies by SARS-CoV-2 variants,^11^^–^^13^ variant-specific symptoms after COVID-19 remain unspecified. Therefore, understanding the characteristics of symptoms by infected strain is crucial to clarify the full spectrum of post-COVID-19 symptoms. Thus, this study aimed to examine the characteristics of variant-specific symptoms after COVID-19 among patients reporting any post-COVID-19 symptoms.

## METHODS

This study was carried out at a single center, which is one of the Designated Medical Institutions for Class II Infectious Disease in Hiroshima Prefecture, Japan. Since the early stage of the COVID-19 outbreak, the hospital has been engaged for COVID-19 patients in various aspects, including COVID-19 diagnosis, inpatient care for individuals with mild-to-moderate disease, and subsequent follow-up for patients regardless of the presence or absence of persistent symptoms. This study aimed to evaluate the characteristics of post-COVID-19 symptoms among epidemic periods, and it is a continuation of our previous publications.^14^^,^^15^

### Study population

There were 385 COVID-19 confirmed patients by polymerase chain reaction test at the hospital who revisited the hospital after COVID-19 recovery for follow-up from September 1, 2020, to March 24, 2022 and provided written informed consent to our study. These patients comprised both those who were admitted to the hospital and those who were quarantined outside the hospital.

Physicians recommended patients visit follow-up consultations based on the patients’ conditions and their preferences. The attendance patterns at follow-up outpatient visits may vary depending on the epidemic period. However, in this study, we specifically focused on individuals with symptoms among the attendees to clarify the characteristics of variant-specific symptoms after COVID-19.

### Survey methods

The participants were asked to complete a set of self-administered questionnaires comprising five sections:

a) basic information, including age, sex, smoking status
b) residual symptoms after COVID-19
c) screening for mood or anxiety disorders using the 6-item Kessler Psychological Distress Scale (K6)
d) screening for impairments in work performance using the Work Functioning Impairment Scale (WFun)
e) experience of COVID-19-related discrimination

K6 scores less than 8 indicated low psychological distress, whereas scores ranging from 8 to 12 indicated moderate psychological distress, and scores ranging from 13 to 24 indicated high psychological distress.^16^ Impairments in work performance were evaluated using WFun scores; scores less than 14 were categorized as normal, whereas scores ranging from 14 to 20 were categorized as mild work performance deficits, scores ranging from 21 to 27 were categorized as moderate work performance deficits, and scores ranging from 28 to 35 were categorized as high work performance deficits.^17^

Patient medical records provided data about COVID-19 onset date, disease severity, place of medical care, medical history, and information on COVID-19 vaccination status at the time of infection. Disease severity was classified into four categories based on the need for oxygen support; mild (ie, no need for supplemental oxygen), moderate (ie, needed for supplemental oxygen), severe (ie, non-invasive mechanical ventilation use), and critical (ie, invasive mechanical ventilation use).^18^

To investigate variant-specific symptoms following COVID-19, participants were grouped into four categories based on their symptom onset date and the monthly distribution of the dominant variant in Hiroshima Prefecture.^19^ These groups were designated as follows:

a) Wild-type period: patients with disease onset between March 2020 and February 2021
b) Alpha period: patients with disease onset between March and June 2021
c) Delta period: patients with disease onset between July 2021 and November 2021
d) Omicron period: patients with disease onset between December 2021 and March 2022

### Statistical analysis

Continuous variables were presented as median and interquartile ranges (IQRs), and the categorical variables as counts and percentages. The Kruskal-Wallis test was used to compare age and the time interval between the onset of COVID-19 and the questionnaire response during each epidemic period. The Chi-square test was used to compare categorical variables. The comparison of K6 scores was restricted to people who had moderate or high psychological distress (K6 score ≥8 points). Similarly, the comparison of WFun scores was restricted to people who had moderate or high work performance deficits (WFun score ≥21 points).

We determined symptom clusters using distances obtained using hierarchical cluster analysis (Ward’s method) and illustrated in the dendrogram. Risk factors and their adjusted odds ratios (AORs) associated with post-COVID-19 symptom clusters were assessed using multivariate analysis. Age and sex were involved as covariates using the forced entry method considering their established strong associations with post-COVID-19 conditions.^20^^,^^21^ Additionally, the epidemic period was included as a covariate in order to evaluate the impact of different infection periods, which was our main objective. The other variables including the severity of COVID-19, smoking status, and comorbidities were entered into the analysis using the stepwise variable selection because the associations with each post-COVID-19 symptom were unclear.^20^^–^^23^ Vaccination and medication history were excluded from the explanatory variables because the vaccines and medications for COVID-19 were just approved in the middle of the survey period of this study, making it impossible to obtain their effects adjusted by the infection period.^24^^,^^25^

Similarly, risk factors and their AORs for psychological distress and impairments in work performance after COVID-19 were assessed. Age, sex, the epidemic period, and the experience of COVID-19-related discrimination were considered as covariates using the forced entry method.^14^^,^^26^ The severity of COVID-19 and smoking status were included in the analysis using the stepwise variable selection.

The statistical analyses were performed using JMP^®^ Version 16 (SAS Institute Japan, Ltd., Tokyo) and *P* < 0.05 was considered statistically significant. As for the stepwise variable selection procedure, *P* < 0.25 was used for both inclusion and exclusion criteria.

### Ethics declarations

This study was approved by the Ethics Committee of Hiroshima University (Approval No. E-2122) and conducted according to the Helsinki Declaration. Furthermore, written informed consent was obtained from each patient before any study procedures.

## RESULTS

**Figure 1.**
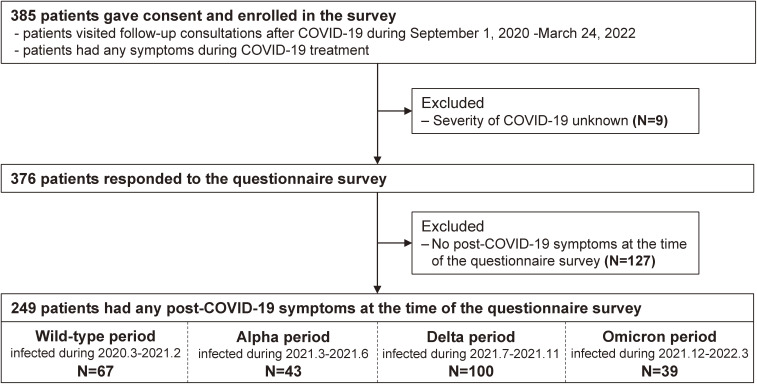
Selection of the study population. This flow chart shows the recruitment and division of patients. The median time interval between the onset of COVID-19 and the questionnaire survey was 23.5 (interquartile range, 20–31) days.

A total of 385 individuals who tested positive for COVID-19 and revisited the hospital for follow-up were enrolled in this study, with the earliest onset of COVID-19 being on April 10, 2020, and the latest on March 7, 2022. Among them, nine patients were excluded for the absence of disease severity, and 127 patients were excluded for the absence of post-COVID-19 symptoms at the time of responding to the questionnaire. Thus, 249 patients with any post-COVID-19 symptoms at the time of responding to the questionnaire were included in the analysis. The questionnaire survey was conducted at a median of 23.5 (IQR, 20–31) days after COVID-19 onset. The number of patients reporting any post-COVID-19 symptoms during the Wild-type, Alpha, Delta, and Omicron periods was 67, 43, 100, and 39, respectively (Figure 1).

### Patient characteristics

Table 1 summarizes the characteristics of patients with any post-COVID-19 symptoms at the time of the questionnaire survey. The median age of the patients was 50 (IQR, 39–58) years, with 26.5%, 53.4%, and 20.1% of patients falling into the age categories of <40, 40–59, and ≥60 years, respectively. This study consisted of 49.4% female participants.

**Table 1.** Patients’ characteristics

			Epidemic periods								

			Wild-type		Alpha		Delta		Omicron		*P* value
			2020.3–2021.2		2021.3–2021.6		2021.7–2021.11		2021.12–2022.3		
	(*N* = 249)		(*N* = 67)		(*N* = 43)		(*N* = 100)		(*N* = 39)		
Age, years, median (IQR) years	50	(39–58)	55	(40–61)	51	(43–59)	48	(36.5–53)	47	(35–63)	0.0136^a^
<40	66	(26.5%)	14	(20.9%)	9	(20.9%)	30	(30.0%)	13	(33.3%)	0.0216^b^
40–59	133	(53.4%)	33	(49.3%)	25	(58.1%)	60	(60.0%)	15	(38.5%)	
≥60	50	(20.1%)	20	(29.9%)	9	(20.9%)	10	(10.0%)	11	(28.2%)	
Sex											
Male	126	(50.6%)	35	(52.2%)	20	(46.5%)	50	(50.0%)	21	(53.8%)	0.9104^b^
Female	123	(49.4%)	32	(47.8%)	23	(53.5%)	50	(50.0%)	18	(46.2%)	
Severity of COVID-19											
Mild	205	(82.3%)	51	(76.1%)	30	(69.8%)	87	(87.0%)	37	(94.9%)	0.0149^b^
Moderate	25	(10.0%)	11	(16.4%)	6	(14.0%)	6	(6.0%)	2	(5.1%)	
Severe	18	(7.2%)	4	(6.0%)	7	(16.3%)	7	(7.0%)	0	(0.0%)	
Critical	1	(0.4%)	1	(1.5%)	0	(0.0%)	0	(0.0%)	0	(0.0%)	
COVID-19 vaccine status											
Not vaccinated	197	(79.1%)	67	(100.0%)	40	(93.0%)	75	(75.0%)	15	(38.5%)	<0.0001^b^
Vaccinated once	19	(7.6%)	0	(0.0%)	3	(7.0%)	16	(16.0%)	0	(0.0%)	
Vaccinated twice	32	(12.9%)	0	(0.0%)	0	(0.0%)	8	(8.0%)	24	(61.5%)	
Unknown	1	(0.4%)	0	(0.0%)	0	(0.0%)	1	(1.0%)	0	(0.0%)	
Place of medical care											
Hospital	227	(91.2%)	59	(88.1%)	40	(93.0%)	99	(99.0%)	29	(74.4%)	<0.0001^b^
Quarantine hotel or home	22	(8.8%)	8	(11.9%)	3	(7.0%)	1	(1.0%)	10	(25.6%)	
Smoking status											
Non smoker	132	(53.0%)	39	(58.2%)	26	(60.5%)	47	(47.0%)	20	(51.3%)	0.3642^b^
Current smoker	34	(13.7%)	7	(10.4%)	3	(7.0%)	19	(19.0%)	5	(12.8%)	
Former smoker	83	(33.3%)	21	(31.3%)	14	(32.6%)	34	(34.0%)	14	(35.9%)	
Comorbidities											
Hypertension	36	(14.5%)	14	(20.9%)	3	(7.0%)	9	(9.0%)	10	(25.6%)	0.0145^b^
Diabetes mellitus	18	(7.2%)	6	(9.0%)	2	(4.7%)	8	(8.0%)	2	(5.1%)	0.6365^b^
COPD	4	(1.6%)	1	(1.5%)	2	(4.7%)	1	(1.0%)	0	(0.0%)	0.3610^b^
Experience of COVID-19-related discrimination											
No	182	(73.1%)	45	(67.2%)	34	(79.1%)	73	(73.0%)	30	(76.9%)	0.5183^b^
Yes	67	(26.9%)	22	(32.8%)	9	(20.9%)	27	(27.0%)	9	(23.1%)	
Duration between COVID-19 onset date and questionnaire survey response, median (IQR) days	23.5	(20–31)	26	(21–59)	25	(21–33)	21	(17–26)	24	(22–29)	<0.0001^a^

COPD, chronic obstructive pulmonary disease; COVID-19, novel coronavirus disease 2019; IMV, invasive mechanical ventilation; IQR, interquartile range; Non-IMV, non-invasive mechanical ventilation.

The data represent the numbers and percentages of relevant patients or median value and IQR. Disease severity was classified into four categories based on the need for oxygen or ventilation support; mild (ie, no need for supplemental oxygen), moderate (ie, needed for supplemental oxygen), severe (ie, non-IMV use), and critical (ie, IMV use).

^a^Kruskal-Wallis test.

^b^Chi-square or Fisher’s exact test.

The majority of patients (82.3%) were mild COVID-19 cases, while 10.0%, 7.2%, and 0.4% were moderate, severe, and critical cases, respectively. At the time of admission, 79.1% of patients were unvaccinated, 7.6% had received one vaccine dose, and 12.9% had received two doses. Most patients (91.2%) received medical care at the hospital, while 8.8% stayed at quarantine hotels or their homes. Differences in age group, COVID-19 severity, COVID-19 vaccination status, and place of medical care, as well as the presence of hypertension, were observed among the four patient groups (*P* < 0.05, Chi-square test).

The median time interval between COVID-19 onset and the questionnaire response was 26 (IQR, 21–59) days, 25 (IQR, 21–33) days, 21 (IQR, 17–26) days, and 24 (IQR, 22–29) days in the Wild-type, Alpha, Delta, and Omicron periods, respectively (*P* < 0.0001, Kruskal-Wallis test).

### Frequently reported symptoms among patients with any post-COVID-19 symptoms

**Figure 2.**
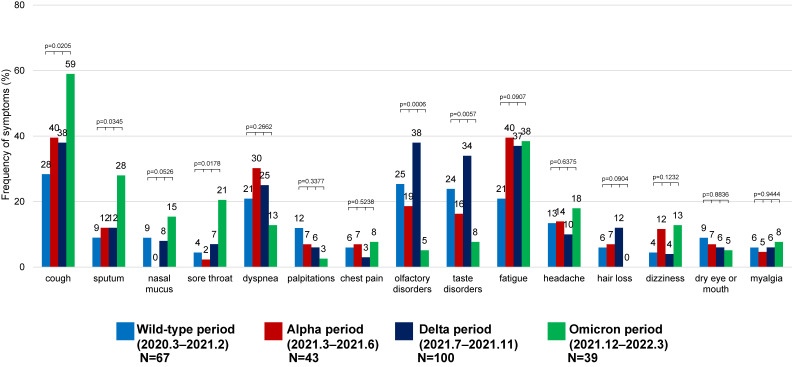
Frequency of persistent symptoms among patients with any post-COVID-19 symptoms

Among patients with any post-COVID-19 symptoms, the most frequently reported symptoms was cough (39.0%), followed by fatigue (33.3%), olfactory disorders (26.1%), taste disorders (24.1%), and dyspnea (22.9%). Furthermore, the frequency of cough, sputum, sore throat, olfactory disorders, and taste disorders differed between epidemic periods (*P* = 0.0205, 0.0345, 0.0178, 0.0006, 0.0057, respectively, Chi-square test in Figure 2).

### Symptom clustering among patients with any post-COVID-19 symptoms

**Figure 3.**
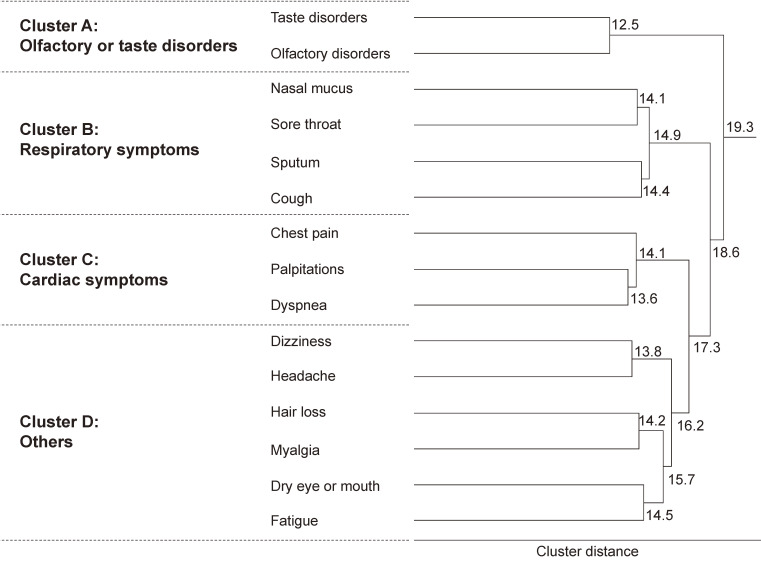
Dendrogram illustrating symptom clustering. Figure shows symptom clusters using distances obtained by hierarchical cluster analysis (Ward’s method).

To cluster symptoms, a hierarchical cluster analysis was performed to identify symptom clusters: Cluster A, which included taste and olfactory disorders; Cluster B, which included nasal mucus, sore throat, sputum, and cough; Cluster C, which included chest pain, palpitations, and dyspnea; and Cluster D which included dizziness, headache, hair loss, myalgia, dry eye or mouth, and fatigue (Figure 3). Odds ratios between each symptom were calculated and presented in eTable 1.

### Risk factors associated with post-COVID-19 symptom clusters

We assessed risk factors associated with post-COVID-19 symptom clusters using multivariate analysis. Since many of the odds ratios between symptoms of Cluster D were not significantly high, only patients with fatigue were included in the analysis from Cluster D.

The results of multivariate logistic regression analysis indicated that olfactory disorders or taste disorders were fewer among patients infected in the Omicron period compared to those infected in the Wild-type period (AOR 0.14; 95% confidence interval [CI], 0.04–0.46; Table 2). In addition, respiratory symptoms were more frequent among patients infected in the Omicron period compared to those infected in the Wild-type period (AOR 3.13; 95% CI, 1.31–7.48). The risk of suffering from post-COVID-19 respiratory symptoms was lower among patients who recovered from severe COVID-19 (AOR for more than mild vs mild, 0.46; 95% CI, 0.22–0.97) but the risk for cardiac symptoms was higher among them (AOR for more than mild vs mild, 2.67; 95% CI, 1.26–5.65). Fatigue was more frequent among patients infected in the Alpha, Delta, or Omicron periods compared to the Wild-type period (AOR 2.65; 95% CI, 1.12–6.26, AOR 2.40; 95% CI, 1.15–5.02, and AOR 2.56; 95% CI, 1.05–6.21, respectively).

**Table 2.** Multivariate analysis results of risk factors for post-COVID-19 symptoms

Factors	a. Olfactory or taste disorders			b. Respiratory symptoms			c. Cardiac symptoms			d. Fatigue		
	(Cluster A)			(Cluster B)			(Cluster C)					
	AOR	95% CI	*P* value	AOR	95% CI	*P* value	AOR	95% CI	*P* value	AOR	95% CI	*P* value
Sex												
Male (ref)	1.00			1.00			1.00			1.00		
Female	1.75	(0.96–3.16)	0.0658	1.25	(0.75–2.11)	0.3932	1.58	(0.86–2.93)	0.1430	0.99	(0.58–1.70)	0.9726
Age, years												
<40 (ref)	1.00			1.00			1.00			1.00		
40–59	0.57	(0.29–1.11)	0.0975	1.53	(0.82–2.85)	0.1788	1.30	(0.62–2.73)	0.4937	1.26	(0.65–2.44)	0.4944
≥60	0.36	(0.14–0.93)	0.0347	1.30	(0.57–2.92)	0.5315	1.57	(0.62–3.96)	0.3420	1.48	(0.65–3.40)	0.3540
Epidemic period												
Wild-type period (2020.3–2021.2, ref)	1.00			1.00			1.00			1.00		
Alpha period (2021.3–2021.6)	0.44	(0.18–1.08)	0.0740	1.06	(0.48–2.33)	0.8881	1.35	(0.56–3.26)	0.5035	2.65	(1.12–6.26)	0.0269
Delta period (2021.7–2021.11)	1.58	(0.80–3.12)	0.1839	0.99	(0.52–1.89)	0.9703	1.35	(0.64–2.84)	0.4340	2.40	(1.15–5.02)	0.0202
Omicron period (2021.12–2022.3)	0.14	(0.04–0.46)	0.0011	3.13	(1.31–7.48)	0.0101	0.93	(0.35–2.52)	0.8911	2.56	(1.05–6.21)	0.0381
Severity of COVID-19												
Mild (ref)	—			1.00			1.00			—		
More than mild	—			0.46	(0.22–0.97)	0.0415	2.67	(1.26–5.65)	0.0103	—		
Smoking status												
Never smoker (ref)	1.00			—			1.00			—		
Current or former smoker	1.43	(0.79–2.59)	0.2339	—			1.85	(1.01–3.40)	0.0478	—		
Hypertension												
No (ref)	1.00			—			—			—		
Yes	1.93	(0.77–4.84)	0.1572	—			—			—		
Diabetes mellitus												
No (ref)	1.00			—			—			1.00		
Yes	0.16	(0.03–0.74)	0.0190	—			—			2.14	(0.78–5.86)	0.1370
COPD												
No (ref)	—			—			—			—		
Yes	—			—			—			—		

AOR, adjusted odds ratio; CI, confidence interval; COPD, chronic obstructive pulmonary disease; COVID-19, novel coronavirus disease 2019.

*n* = 249.

Response variable: a; the presence or absence of olfactory or taste disorders, b; the presence or absence of cough, sputum, nasal mucus, or sore throat, c; the presence or absence of dyspnea, palpitations, or chest pain, d; the presence or absence of fatigue.

Explanatory variables (forced entry): sex, age, and epidemic period, Explanatory variables (stepwise variable selection): severity of COVID-19, smoking status, hypertension, diabetes mellitus, and COPD.

### Psychological distress among patients with any post-COVID-19 symptoms

**Figure 4.**
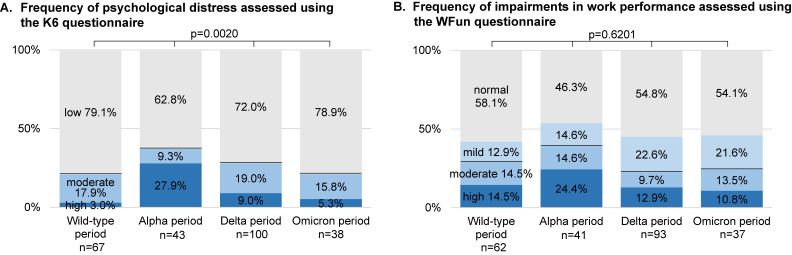
Frequency of psychological distress and impairments in work performance. **A**) total scores <8 were classified as low psychological distress, and 8–12 were classified as moderate psychological distress, and 13–24 were classified as high psychological distress. The intensity of the color relates to the severity of psychological distress. The comparison of K6 scores was restricted to people who had moderate or high psychological distress (K6 score ≥8 points) **B**) total scores <14 were classified as normal, and scores ≥14 suggested possible impairments in work performance (14–20, mild; 21–27, moderate; and 28–35, high). The intensity of the color relates to the severity of impairments in work performance. The comparison of WFun scores was restricted to people who had moderate or high work performance deficits (WFun score ≥21 points). The subjects with incomplete responses were not included in the analysis.

The distribution of patients with moderate or high psychological distress (defined by K6 score ≥8 points) varied across the Wild-type, Alpha, Delta, and Omicron periods, with respective percentages of 20.9%, 37.2%, 28.0%, and 21.1% (Figure 4A). This indicates a significant difference in K6 score distribution among epidemic periods. Multivariate analysis revealed that infection during the Alpha period (AOR for Alpha vs Wild-type, 2.55; 95% CI, 1.02–6.35), experiences of COVID-19-related discrimination (AOR with experience vs without experience, 3.14; 95% CI, 1.64–6.00), and severe COVID-19 cases (AOR for more than mild vs mild, 2.57; 95% CI, 1.13–5.85; Table 3) increased the risk of psychological distress after COVID-19. Conversely, individuals aged ≥60 years had a significantly lower risk of psychological distress than those under 40 years old (AOR for ≥60 vs <40 years, 0.34; 95% CI, 0.12–0.95).

**Table 3.** Multivariate analysis results of risk factors for psychological distress and impairments in work performance after COVID-19

Factors	a. K6 score ≥8 points			b. WFun score ≥21 points		
	AOR	95% CI	*P* value	AOR	95% CI	*P* value
Sex						
Male (ref)	1.00			1.00		
Female	1.68	(0.91–3.12)	0.0976	1.71	(0.93–3.15)	0.0862
Age, years						
<40 (ref)	1.00			1.00		
40–59	0.59	(0.29–1.18)	0.1356	0.63	(0.31–1.26)	0.1902
≥60	0.34	(0.12–0.95)	0.0395	0.71	(0.28–1.77)	0.4571
Epidemic period						
Wild-type period (2020.3–2021.2, ref)	1.00			1.00		
Alpha period (2021.3–2021.6)	2.55	(1.02–6.35)	0.0451	1.82	(0.76–4.38)	0.1809
Delta period (2021.7–2021.11)	1.60	(0.73–3.52)	0.2445	0.73	(0.34–1.59)	0.4295
Omicron period (2021.12–2022.3)	1.27	(0.44–3.66)	0.6639	0.82	(0.31–2.15)	0.6823
Experience of COVID-19-related discrimination						
No (ref)	1.00			1.00		
Yes	3.14	(1.64–6.00)	0.0005	2.23	(1.17–4.27)	0.0149
Severity of COVID-19						
Mild (ref)	1.00			—		
More than mild	2.57	(1.13–5.85)	0.0243	—		
Smoking status						
Never smoker (ref)	—			—		
Current or former smoker	—			—		

CI, confidence interval; COVID-19, novel coronavirus disease 2019; AOR, adjusted odds ratio.

The number of subjects: a; *n* = 248, b; *n* = 233.

Response variable: a; K6 score ≥8 points, b; Response variable: WFun score ≥21 points.

Explanatory variables (forced entry): sex, age, epidemic period, and experience of COVID-19-related discrimination, Explanatory variables (stepwise variable selection): severity of COVID-19 and smoking status.

### Impairments in work performance among patients with any post-COVID-19 symptoms

The percentages of patients who had moderate or high impairments in work performance (WFun score ≥21 points) were recorded as 29.0%, 39.0%, 22.6%, and 24.3% during the Wild-type, Alpha, Delta, and Omicron periods, respectively (Figure 4B). The distribution of the WFun score was not significantly differed across the epidemic periods. Multivariate analysis revealed that COVID-19-related discrimination was a potential risk factor for impairments in work performance following COVID-19 (AOR with experience vs without experience, 2.23; 95% CI, 1.17–4.27; Table 3). However, there was no significant association observed between impairments in work performance and sex, age, or infected period.

## DISCUSSION

The objective of this hospital-based questionnaire survey was to investigate the appearance of symptoms in the different dominant periods of COVID-19 among patients with any persistent symptoms. There was limited information on post-COVID-19 symptoms dividing the VOC epidemic periods.^27^^–^^30^

This study result indicated that olfactory disorders and taste disorders were common post-COVID-19 symptoms, and they were likely to co-occur. Previous studies suggested that individuals with taste disturbances often have a normal sense of taste, indicating that their taste function may be impaired due to olfactory disorders.^31^ The multivariate logistic regression analysis indicated that olfactory or taste disorders were fewer among patients infected with the Omicron variant compared to the Wild-type variant. As mentioned in recent studies, the loss of smell function is closely related to D614G mutation.^32^^,^^33^ Omicron also has a D614G mutation, but due to new mutations of spike protein, which introduced more alkaline and hydrophobic stretches than the Wild-type and Delta variants, may reduce infection of the olfactory epithelium. This could potentially contribute to the sparing of olfaction in patients infected with the omicron variant.^34^

On the other hand, respiratory symptoms were more common in patients infected during the Omicron period compared to those infected during the Wild-type period. It can be explained by the fact that the Wild-type variant remarkedly causes lower respiratory tract infection, while the Omicron variant causes upper respiratory tract infection.^35^^–^^37^ In addition, those infected with the Omicron variant are more likely to have common cold symptoms, such as sore throat, cough, fatigue, nasal mucus, and headache during COVID-19 infection.^13^^,^^38^^,^^39^ The specific symptoms after COVID-19 may depend on the SARS-CoV-2 variant.

Cluster analysis showed that dyspnea was more likely to co-occur with chest pain and palpitations than with respiratory symptoms such as nasal mucus, sputum, or cough symptoms. Despite having normal pulmonary function and exercise tolerance, patients with post-COVID-19 may experience dyspnea.^40^ The American Thoracic Society has suggested that dyspnea is a result of various physiological, psychological, social, and environmental factors interacting with each other.^41^ Our study found that the frequency of cardiac symptoms, including dyspnea, did not vary across the four epidemic periods, and the risk factors were the severity of COVID-19 and smoking history.

Persistent post-COVID-19 symptoms have been linked to a decrease in the quality of life and the development of anxiety, depression, and sleeping disorders.^42^^–^^44^ In our study, we found that the frequency of high psychological distress, as measured by K6 scores, was higher during the alpha variant periods (3.0%, 27.9%, 9.0%, and 5.3% in Wild-type, Alpha, Delta, and Omicron periods, respectively) compared to the 10.8% reported in a previous study of Japanese employees.^16^ Our research findings also revealed a strong association between contracting COVID-19 during the Alpha period, as well as COVID-19-related discrimination, and experiencing psychological distress. It is possible that the variation in K6 scores may be influenced by stigma or prejudice related to COVID-19 infection, or the socioeconomic status of individuals at the time of responding to the questionnaire.

The proportions of individuals who scored over 20 in the WFun assessment in each group (29.0%, 39.0%, 22.6%, and 24.3% in the Wild-type, Alpha, Delta, and Omicron periods, respectively) were found to be either comparable to or greater than the reported 20% prevalence in the general working population of Japan.^17^ This suggests that individuals around post-COVID-19 patients who have resumed social activities should provide support and consider their mental health and work performance.

However, this study has some limitations. First, this study was conducted at a single center with insufficient sample size, and few severe cases were included. Although the hospital is the main center for COVID-19 treatment in Hiroshima Prefecture, the results of this study need to be validated through multicenter surveys. Additionally, the admission rate in COVID-19 patients could be influenced by the epidemic period. However, this study included not only patients who were admitted to the hospital but also patients who were quarantined outside the hospital, the differences in admission rate have limited impact on this study examining the characteristics of post-COVID-19 symptoms by epidemic period. Second, we cannot calculate the prevalence of post-COVID-19 symptoms in this study since not all patients after COVID-19 recovery visited the follow-up consultations and the prevalence may vary depending on the epidemic period. However, we specifically focused on individuals with symptoms among the attendees of follow-up consultations to clarify the characteristics of variant-specific symptoms after COVID-19, so that the attendance rate for follow-up consultation visits has limited impact on the results of this study. Third, we could not test specific SARS-CoV-2 strains in each subject, instead, patients were categorized into groups based on the epidemic periods of each strain as reported in Hiroshima.^19^ Fourth, we conducted this study using self-administered questionnaires, which introduces the potential for both underestimation and overestimation of the impact of post-COVID-19 symptoms. Fifth, this study questionnaire did not include an assessment of symptom severity or the impact of post-COVID-19 symptoms on the daily lives of patients. Sixth, this study was conducted in Japanese only and did not examine in the other races. Seventh, the period between the onset of COVID-19 and the administration of the questionnaires in this study was shorter than in other studies. Even with such limitations, this long-running study which used the same questionnaire throughout and asked about symptoms at the time of questionnaire response has provided valuable results revealing the characteristics of symptoms after COVID-19 by variants without recall bias.

In conclusion, this study revealed the post-COVID-19 symptoms varied by the virus variants, and the risk of suffering from post-COVID-19 symptoms depends on the severity of the disease the patients experienced. In order to achieve a comprehensive understanding of the characteristic of post-COVID-19 symptoms, it is necessary to conduct a multicenter, long-term survey that investigates the symptom severity and their consequential effects on individuals’ quality of life.

## SUPPLEMENTARY MATERIAL

eTable 1. Associations among post-COVID-19 symptoms
