# Supplementary material for: Variant-specific Symptoms After COVID-19: A Hospital-based Study in Hiroshima
Source: J Epidemiol. 2024 May 5;34(5):238–46. doi: 10.2188/jea.JE20230103 (PMC10999520; doi:10.2188/jea.JE20230103)
Supplement: Supplementary file 1 [file je-34-238-s001.pdf]

**eTable 1.** Associations among post-COVID-19 symptoms

| | | Cluster A: Olfactory or taste disorders | | Cluster B: Respiratory symptoms | | | | Cluster C: Cardiac symptoms | | | Cluster D: Others | | | | | |
|---|---|---|---|---|---|---|---|---|---|---|---|---|---|---|---|---|
| | | Taste disorders | Olfactory disorders | Nasal mucus | Sore throat | Sputum | Cough | Chest pain | Palpitations | Dyspnea | Dizziness | Headache | Hair loss | Myalgia | Dry eye or mouth | Fatigue |
| Cluster A: Olfactory or taste disorders | Taste disorders (n=60) | | 6.00 | 0.53 | 1.50 | 0.64 | 0.23 | 0.56 | 0.89 | 1.31 | 0.97 | 1.06 | 0.83 | 1.63 | 1.80 | 1.34 |
| | Olfactory disorders (n=65) | 33 | | 0.69 | 1.01 | 0.24 | 0.30 | 0.22 | 0.80 | 0.70 | 1.19 | 1.13 | 1.73 | 1.45 | 2.10 | 0.70 |
| Cluster B: Respiratory symptoms | Nasal mucus (n=20) | 3 | 4 | | 5.12 | 4.03 | 1.63 | 0.95 | 0.66 | 1.13 | 0.70 | 2.49 | 2.35 | 4.95 | 2.71 | 1.37 |
| | Sore throat (n=19) | 6 | 5 | 5 | | 3.33 | 3.77 | 1.01 | 1.57 | 2.69 | 1.69 | 3.62 | 1.47 | 3.41 | 4.45 | 2.39 |
| | Sputum (n=34) | 6 | 3 | 7 | 6 | | 2.56 | 1.16 | 0.35 | 1.49 | 2.07 | 1.56 | 0.73 | 0.44 | 2.07 | 1.96 |
| | Cough (n=97) | 10 | 13 | 10 | 13 | 20 | | 0.68 | 0.58 | 0.53 | 1.10 | 1.26 | 0.54 | 0.37 | 0.85 | 0.77 |
| Cluster C: Cardiac symptoms | Chest pain (n=13) | 2 | 1 | 1 | 1 | 2 | 4 | | 7.05 | 6.11 | 0.00 | 1.25 | 4.13 | 3.12 | 1.15 | 1.77 |
| | Palpitations (n=18) | 4 | 4 | 1 | 2 | 1 | 5 | 4 | | 6.32 | 0.79 | 2.91 | 2.69 | 0.91 | 1.80 | 2.71 |
| | Dyspnea (n=57) | 16 | 12 | 5 | 8 | 10 | 16 | 8 | 11 | | 1.44 | 2.69 | 2.10 | 1.24 | 3.32 | 1.64 |
| Cluster D: Others | Dizziness (n=17) | 4 | 5 | 1 | 2 | 4 | 7 | 0 | 1 | 5 | | 5.80 | 1.69 | 2.25 | 1.93 | 4.07 |
| | Headache (n=32) | 8 | 9 | 5 | 6 | 6 | 14 | 2 | 5 | 13 | 7 | | 1.30 | 2.68 | 1.50 | 1.93 |
| | Hair loss (n=19) | 4 | 7 | 3 | 2 | 2 | 5 | 3 | 3 | 7 | 2 | 3 | | 5.31 | 1.69 | 1.18 |
| | Myalgia (n=15) | 5 | 5 | 4 | 3 | 1 | 3 | 2 | 1 | 4 | 2 | 4 | 4 | | 3.93 | 3.24 |
| | Dry eye or mouth (n=17) | 6 | 7 | 3 | 4 | 4 | 6 | 1 | 2 | 8 | 2 | 3 | 2 | 3 | | 3.11 |
| | Fatigue (n=83) | 23 | 18 | 8 | 10 | 16 | 29 | 6 | 10 | 24 | 11 | 15 | 7 | 9 | 10 | |

COVID-19, novel coronavirus disease 2019.
The data below the diagonal line represent the numbers of each pair of post-COVID-19 symptoms among the subjects, while the data above the diagonal line represent the odds ratio to co-occur the symptoms. Highlighted cells have significantly higher or lower odds ratio (yellow color: $P<0.05$, red color: $P<0.01$, using the Chi-square or the Fisher's exact test).
